# Evidence of the CH···O HydrogenBonding in Imidazolium-Based Ionic Liquids from Far-Infrared Spectroscopy Measurements and DFT Calculations

**DOI:** 10.3390/ijms22116155

**Published:** 2021-06-07

**Authors:** Oriele Palumbo, Adriano Cimini, Francesco Trequattrini, Jean-Blaise Brubach, Pascale Roy, Annalisa Paolone

**Affiliations:** 1 CNR-ISC, U.O.S. La Sapienza, Piazzale A. Moro 5, 00185 Rome, Italy; oriele.palumbo@roma1.infn.it (O.P.); cimini.1233261@studenti.uniroma1.it (A.C.); annalisa.paolone@roma1.infn.it (A.P.); 2 Physics Department, Sapienza University of Rome, Piazzale A. Moro 5, 00185 Rome, Italy; 3 Synchrotron SOLEIL, AILES Beamline, L’Orme des Merisiers Saint-Aubin, BP 48, CEDEX, 91192 Gif-sur-Yvette, France; jean-blaise.brubach@synchrotron-soleil.fr (J.-B.B.); pascale.roy@synchrotron-soleil.fr (P.R.)

**Keywords:** ionicliquids, hydrogen bonding, infrared spectroscopy, DFT calculations

## Abstract

Knowledge of all the intermolecular forces occurring in ionic liquids (ILs) is essential to master their properties. Aiming at investigating the weaker hydrogen bonding in aprotic liquids, the present work combined computational study and far-infrared spectroscopy on four imidazolium-based ILs with different anions. The DFT calculations of the ionic couples, using the ωB97X-D functional and considering both the empirical dispersion corrections and the presence of a polar solvent, show that, for all samples, the lowest energy configurations of the ion pair present H atoms, directly bound to C atoms of the cation and close to O atoms of the anion, capable of creating moderate to weak hydrogen bonding with anions. For the liquids containing anions of higher bonding ability, the absorption curves generated from the calculated vibrational frequencies and intensities show absorption bands between 100 and 125 cm^−1^ corresponding to the stretching of the hydrogen bond. These indications are in complete agreement with the presently reported temperature dependence of the far-infrared spectrum, where the stretching modes of the hydrogen bonding are detected only for samples presenting a moderate interaction and become particularly prominent at low temperatures. Moreover, from the analysis of the infrared spectra, the occurrence of various phase transitions as a function of temperature was detected, and the difference in the average energy between the H-bonded and the dispersion-governed molecular configurations was evaluated.

## 1. Introduction

Ionic liquids (ILs) are more than ever attracting great attention in fundamental as well as in applicative science owing to the possibility of tuning their remarkable properties by a proper choice of the composing ions or even by changing the alkyl chain length [1,2] or the functional groups within the composing ions [3]. In addition to modify the ionic steric hindrance, these selections mainly introduce a variation of the relative strengths of Coulomb interactions, hydrogen bonding, and dispersion forces [4]. Therefore, full knowledge of all the intermolecular forces occurring in ILs is fundamental to improve their performance by a proper selection of the ions.

ILs are salts that display a very low melting point, whose interionic interactions are mainly governed by Coulombic forces: however, when cations contain H atoms and anions possess lone pairs, the cations can act as H-bond donors and the anions as H-bond acceptors [5]. In particular, protic ionic liquids (PILs), composed through the neutralization reaction of a Brønsted acid with a Lewis base, possess exchangeable protons and strong hydrogen bonds [6,7,8], where the H atom is often covalently bound to the heavy cation carrying the formal charge [5,9]. In aprotic ILs, instead, weak H bonds are usually formed with C–H group of the cation as the primary H-bond donor unit [5]. Since the H atom is directly linked to a carbon atom, the bond to the O atom of the anion has a reduced strength, but it is widely accepted that this link can nevertheless be considered as a true hydrogen bonding [5,10]. With regard to the anions, a large range of potential H-bond acceptors are reported with different H-bonding ability, from strong H-bond acceptors such as acetate, chloride [5] or methanesulfonate (MS) [11], to very weak H-bond acceptors such as bis(trifluoromethanesulfonyl)imide (TFSI) [5]. In particular, in imidazolium-based ILs, the occurrence of a hydrogen bond between the C-H ring and the oxygen of the TFSI anion was reported [11,12,13].

Even if H-bonding in ILs is typically established between a cation and an anion, it has been recently reported [14,15] between cations in the bulk phase of hydroxy-functionalized ILs leading to the formation of H-bonded cation clusters. Moreover, this interaction can be tuned via the coordination strength of the anion, the charge delocalization of the cation, and the length of the hydroxyalkyl chain [15,16,17].

Vibrational spectroscopy, frequently combined with DFT calculations, has been largely used to study the occurrence of H-bonding both in protic and aprotic ILs [5,10,18], and infrared spectroscopy in particular has emerged as the method of choice for studying its spectroscopic properties [19]. The studies based on far-infrared (FIR) spectroscopy, even though requiring demanding experimental setup, have provided interesting results on the competing effects of hydrogen bonding [20] and dispersion interactions. Indeed, the exploitation of the FIR range is especially meaningful for PILs because their strong directional hydrogen bonding [8,21,22] gives rise to a well isolated and narrow peak around 150–170 cm^−1^, similarly to what is detected in ice at ~100 K, with two bands around 230 and 160 cm^−1^ corresponding to intermolecular hydrogen bond stretching modes, with motions in different direction [23]. In particular, the spectra of PILs containing an ammonium cation with a single proton-donor group (N–H) display an isolated vibrational band between 150 and 170 cm^−1^ assigned to the stretching of the hydrogen bond between the cationic NH and the anion [8,11,21,24,25,26]. Moreover, for these systems, the combined use of FIR spectroscopy and computational approaches provided a quantitative description of the competition between hydrogen bonding and dispersion forces [8,21,24,25,26].

**Figure 1:**
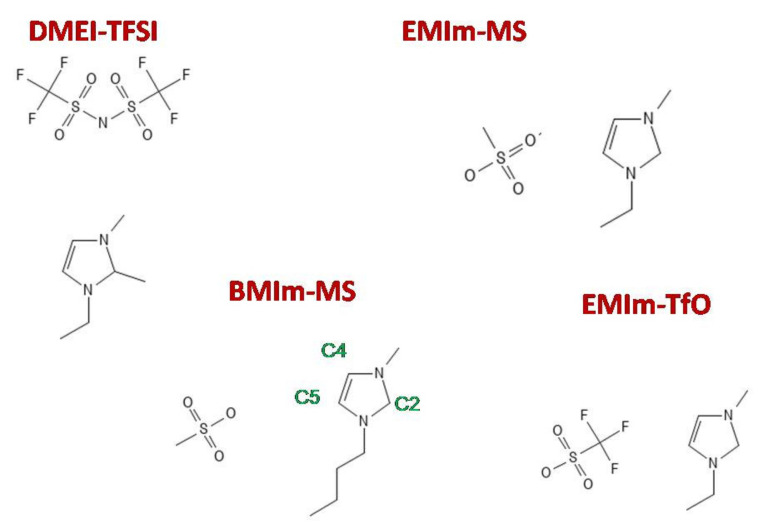
Scheme of the cation and anion composing the investigated ionic liquids with labels of the C atoms of the imidazolium ring.

FIR spectroscopy [25,27,28,29,30,31] has also been exploited to probe intermolecular interactions in aprotic ILs, with contrasting results, however. Several studies [25,28,29,30] attributed the broad band detected below 150 cm^−1^ in the FIR spectrum of imidazolium-based ILs to the superposition of the bending and stretching vibrational modes of the hydrogen bonding between the anion and the C-H group of the imidazolium ring, involving the carbon atom, C(2), located between the two nitrogen atoms [25,30] (see Figure 1 for identification of C(2)). The frequency of this mode, which shifted to higher wavenumbers with increasing ionic strength of the anion, [25,29], can be correlated to the average binding energies of the ionic liquid, obtained by means of DFT calculations on ionic couples [29]. Conversely, other authors [27,28], calculating the optical constants from the FIR absorbance, attributed the broad band measured at low frequencies to interionic motions [28]. In particular they pointed that, for ILs having weak coordinating anions, this band is practically unchanged even if the acidic C(2)-H imidazolium bond is methylated or when the imidazolium cation is substituted by tetra-alkyl ammonium or pyrrolidinium cations, thus implying that it is a general feature of ILs, caused by the relative translational and librational motions of the ions without any hydrogen bond character [27,28].

A similar debate also involved results obtained in the mid-infrared range (MIR), where the hydrogen bonding can be detected through a detailed analysis of the C-H stretching modes. Indeed, the redshift of the donors (X-H) stretching frequencies generally indicates that the X-H bond is weakened, concomitantly with the elongation of the X-H distance, upon the formation of the H bonding with the acceptor group [5,10,27,32]. In particular, the comparison of measured and calculated spectra in the frequency range between 3000 and 3300 cm^−1^ allowed the attribution of the observed lines to the stretching modes of the C(4/5)- and of the C(2)-H [11,29] bonds of the imidazolium ring (see Figure 1 for the meaning of the labels). The latter showed the largest redshift, indicating a stronger interaction with the anion. It must be pointed out that these shifts are significantly smaller in magnitude than their classical O-H···O counterparts [10]. An alternative approach, based on isotopic substitution, allowed to ascribe the redshift of the C-H stretching frequencies of the imidazolium ring to the occurrence of Fermi resonance interactions with the overtones and combination of in-plane ring modes [27,28].

A direct measurement of the strength of the hydrogen bonding in imidazolium-based ILs was also obtained by the analysis of the anion-dependent differential ^1^H NMR shifts [33,34,35]. Most NMR studies used ILs diluted in organic solvents, which havean influence on the NMR shifts [35,36]; however, low viscosity ILs can be studied in pure form by NMR spectroscopy, thus allowing a direct and easier interpretation of the measured spectra [35]. Moreover, a combination of theoretical and experimental methods suggested that both the IR and the NMR spectroscopic results show a similar electronic perturbation attributed to hydrogen bonding [37].

Here, we report a combined computational- and temperature-dependent FIR spectroscopy study on four imidazolium-based ILs with different anions. In particular, we investigated two liquids combining the same cation with two different anions (1-ethyl-3-methylimidazolium trifluoromethanesulfonate, EMI-TfO, and 1-ethyl-3-methylimidazolium methanesulphonate, EMI-MS) and two ILs possessing a common anion with different cations (EMI-MS and 1-butyl-3-ethylimidazolium methanesulphonate, BMI-MS). The comparison of the first two systems aimed at evaluating the effects of anions with different attracting ability, while the other two allow assessment of the effects of different cationic side groups. A fourth ionic liquid (1-ethyl-2,3-dimethylimidazolium bis(trifluoromethanesulfonyl)imide, DMEI-TFSI) with the low attracting TFSI anion and a cation of the imidazolium family was also studied for comparison. For each system, we investigated the temperature evolution of the spectra measured in the far IR range and compared the spectra to the DFT computational simulation for the ionic couple performed using the ωB97X-D functional. The simulation also took into account the empirical dispersion corrections and the presence of a polar solvent. This combined approach allowed the detection of moderate hydrogen bonding, overcoming previous contrasting results. Moreover, from this analysis, the difference in the average energy between the H-bonded and the dispersion-governed configurations was evaluated.

## 2. Materials and Methods

The ionic liquids used for the present study, 1-ethyl-2,3-dimethylimidazolium bis(trifluoromethanesulfonyl)imide (labeled as sample DMEI-TFSI), 1-ethyl-3-methylimidazolium trifluoromethanesulfonate (labeled as sample EMI-TfO), 1-ethyl-3-methylimidazolium methanesulphonate (labeled as sample EMI-MS), and 1-butyl-3-ethylimidazolium methanesulphonate (labeled as sample BMIM-MS), were purchased from Iolitec, Heilbronn, Germany (samples DMEI-TFSI and EMI-TfO, having a purity higher than 90%) and from Sigma Aldrich, St. Louis, MO, USA (samples EMI-MS and BMIM-MS, with a purity ≥95%). A schematic picture of the samples is reported in Figure 1.

All ILs samples were stored in a glove box and were dried in a vacuum better than 10^−5^ mbar for 48 h to minimize their water content just before measurements.

Infrared spectroscopy was performed using a Bruker IFS125 HR Fourier transform interferometer (Billerica, MA, USA) at the AILES beamline of the SOLEIL Synchrotron. The spectra were recorded in the far-infrared range using a Si coated 6-micron-thick mylar beamsplitter and a bolometer; 100 scans measured at a resolution of 1 cm^−1^ were averaged for both the transmission through the empty cell and the sample containing cell. For the latter, thin layers of ILs were placed between the diamond windows of the vacuum-tight cell. The transmission was calculated using the spectrum of the empty cell as a reference and was then converted into absorbance spectrum. The samples were cooled down to 120 K by means of a Cryomec cryostat (Syracuse, NY, USA) at a temperature rate of 5 Kmin^−1^, and the data were collected both upon cooling and heating in the range between this minimum temperature and 330 K.

The lowest energy configuration of each of the four ionic couples was investigated by means of DFT calculations using Spartan software [38,39] (Wavefunction, Irvine, CA, USA). Each ionic couple (IC) was constructed, and a search for all conformers was performed by means of molecular mechanics (force field MMFF94). The search was performed by systematic rotations of the flexible bonds by 120°. A list of possible non-duplicate conformers was then generated for each IC. There were 2 for EMI-TfO and EMI-MS, 19 for BMIM-MS, and 18 for DMEI-TFSI. All conformers were subject to geometry optimization by DFT calculations by means of the ωB97X-D functional and considering the presence of a polar solvent (ε_r_ = 37.22) and empirical dispersion forces [40]. The 6-31G** basis set was employed in all calculations. Subsequently, a calculation of the vibrational frequencies and intensities was performed. Only the lowest energy conformer that possessedall positive frequencies isreported in the present paper.

The choice of the particular combination of functional dispersion forces and electrostatic mediumwas motivated by our previous experimental and computational investigation of the prototypes of protic ionic liquids, diethyl-methyl-ammonium methanesulfonate(DEMA-MS) and diethyl-methyl-ammonium trifluoromethanesulfonate(DEMA-TfO) [8]. The absorbance spectra calculated by means of various functional (B3LYP, B3LYP-D, ωB97X-D) in the gas phase or in a polarizable medium were compared withthe experimental data. Among all these functionals, the best performing concerning the hydrogen bonding bands was ωB97X-D with the inclusion of empirical dispersion forces and a polar medium. The polar medium was included by the PCM solvation scheme.The same combination of functional and basis set was previously used to investigate the infrared spectrum of EMI-TfO in the spectral range 200–1800 cm^−1^, providing a satisfactory agreement [20].

In the present study, the infrared vibration frequencies and intensities were calculated for the lowest energy configuration presenting only positive vibration frequencies. Subsequently, for the purpose of comparing the experimental infrared spectrum with the simulations, we calculated infrared spectra by summing Gaussian curves centered at each calculated vibration frequency, with a 10 cm^−1^ line width and with intensity proportional to the calculated one.

## 3. Results

### 3.1. Computational Results

**Figure 2:**
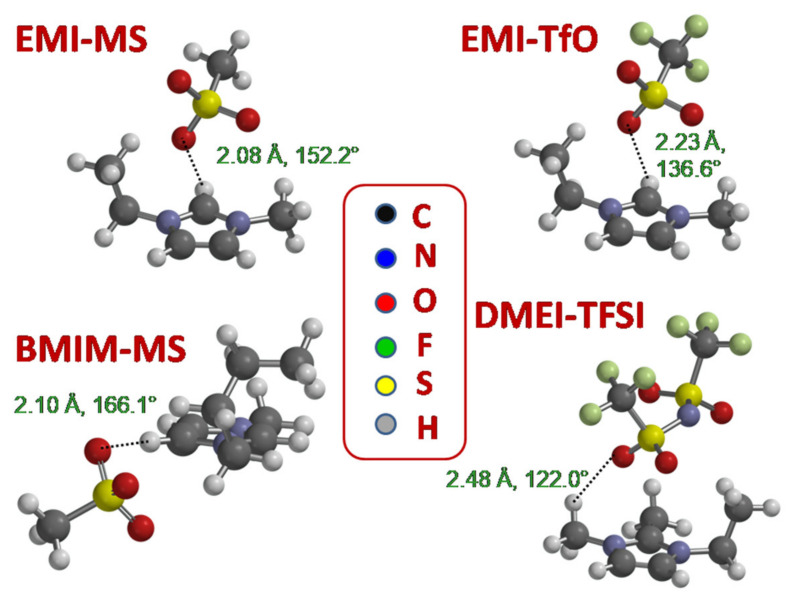
Lowest energy ionic couples composing the four ionic liquids, calculated by DFT using the ωB97X-D functional and considering the presence of a polar solvent (ε_r_ = 37.22); in all cases the 6-31G** basis set was employed. The dotted lines show the closest H-O bonds for each.

Figure 2 reports the lowest energy structure of the four ion pairs, EMI-MS, EMI-TfO, BMIM-MS, and DMEI-TFSI, for which only positive vibrational frequencies were determined (reported in Table S1 of the Supplementary Materials). The configurations were calculated by means of the ωB97X-D functional and considering the presence of a polar solvent (ε_r_ = 37.22) and empirical dispersion forces.

For DMEI-TFSI, the lowest energy configuration of the ion pair involves the TFSI anion in its trans-configuration, which is known to be more stable than the cis-configuration [41]. The other two anions, MS and TfO, each possesses one unique conformer.

It can be noted that in all pairs, one H atom of the cation directly bound to a C atom is located close to an O atom of the anion (at a distance shorter than 2.50 Å), forming a hydrogen bond. For the two liquids with the EMI cation, the H atom which is closest to the anion is linked to the cationic C between the two nitrogen atoms (usually called C2 position), while for the liquid with BMIM, the involved H belongs to the ring (C4 or C5 positions) and finally, for the DMEI-TFSI, the H atom is linked to a C atom from the methyl side group. Table 1 reports the closest H-O distance and the angles formed between C, H, and the nearest O atom of the anion.

The distances between the closest H atom of the cation and O atom of the anion atoms are typical of weak to moderate hydrogen bonding [42]; in particular, the values obtained for EMI-MS, EMI-TfO, and BMIM-MS range between 2.08 and 2.23 Å: EMI-MS shows the shortest distance of 2.08 Å, suggesting the strongest bond. Moreover, the angles formed by the CHO atoms range between 136 and 166° for EMI-MS, EMI-TfO, and BMIM-MS, while it is 122° for DMEI-TFSI; this implies that for the latter ionic couple, the H-O bond with the anion is less aligned with the CH bond of the cation.

### 3.2. Experimental Results

#### 3.2.1. Occurrence of Phase Transitions

A detailed examination of the temperature dependence of the IR spectra of the samples can provide indications about the possible phase transitions taking place in the measured temperature range as well as the changes occurring in the intermolecular interactions. The occurrence of a phase transition is usually indicated by an abrupt change in the temperature dependence evolution of lines, which are due to single ion vibrations, while changes in intermolecular interactions are detectable in the evolution of bands below 200 cm^−1^.

**Figure 3:**
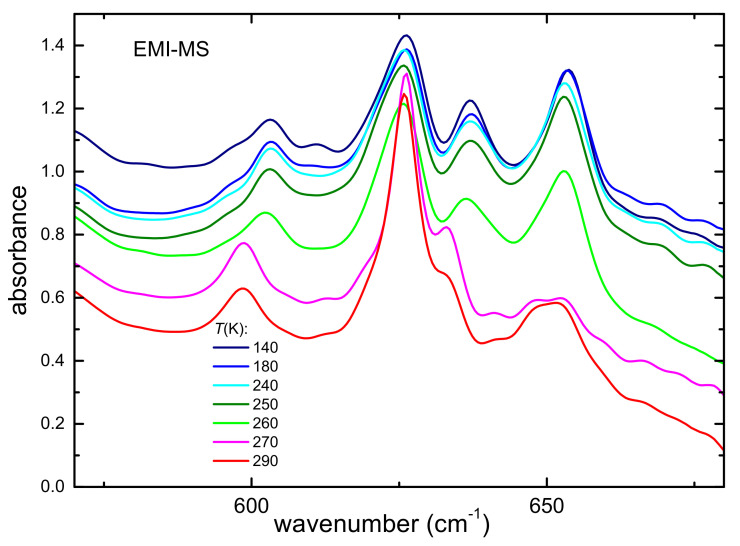
Absorbance of EMI-MS between 550 and 700 cm^−1^ measured on heating. The curves are vertically shifted for reasons of clarity.

The absorbance spectrum of sample EMI-MS measured on heating up to 300 K in the frequency range between 550 and 700 cm^−1^, where the intramolecular vibrations of single ions are observed, is displayed in Figure 3.

At the lowest temperature, the spectrum shows several absorption lines around 605, 626, 635, and 658 cm^−1^. On heating at 260 K, the line at 635 cm^−1^ disappears, and the one at 658 cm^−1^ suddenly broadens. Concomitantly, the lines at 605 and 626 cm^−1^ also present an abrupt shift of their peak frequency. These changes are likely due to the melting of the sample around 270 K, in agreement with previous DSC measurements [43].

**Figure 4:**
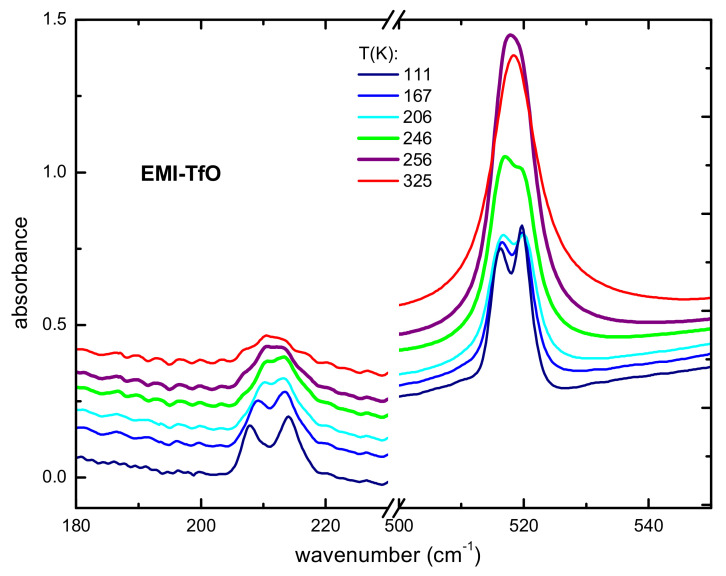
Absorbance of EMI-TfO measured on heating between 180 and 550 cm^−1^. The curves are vertically shifted for reasons of clarity.

The absorbance spectrum of sample EMI-TfO measured between 180 and 550 cm^−1^ on heating between 110 and 290 K is reported in Figure 4.

At 110K, two pairs of narrow peaks are well detectable, one with peak frequencies at 205 and 215 cm^−1^, and the other one with peak frequencies between 515 and 520 cm^−1^, which can be safely attributed to the anion vibrations, in agreement with previous computational results obtained for the isolated anion [8,44]. On heating above 250 K, both doublets merge into two broad bands, respectively at 210 and 515 cm^−1^, signaling the occurrence of a solid–liquid transition, which was already evidenced for this IL by differential scanning calorimetry and mid-infrared spectroscopy measurements [44,45].

**Figure 5:**
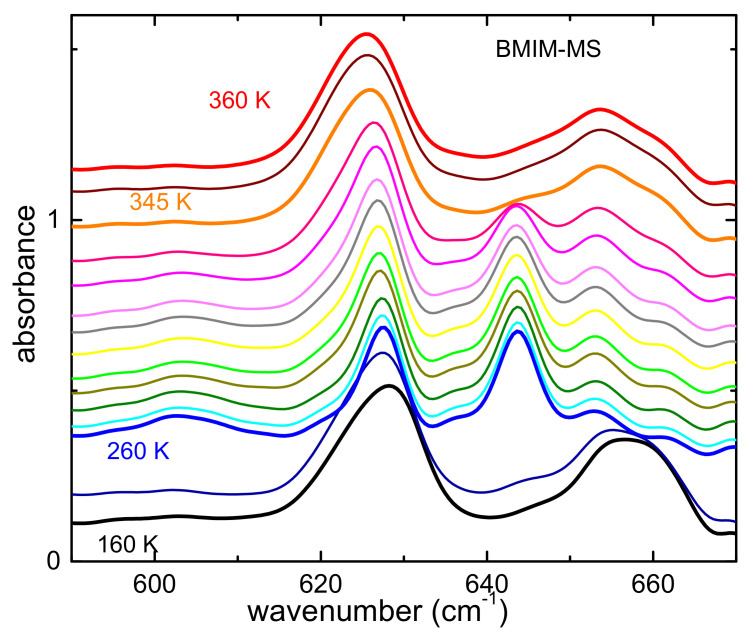
Absorbance of BMIM-MS measured on heating between 600 and 680 cm^−1^. The curves are vertically shifted for reasons of clarity.

The temperature dependence of the FIR spectrum of sample BMIM-MS between 600 and 680 cm^−1^ is reported in Figure 5.

In the reported frequency range, at the lowest temperature, the spectrum of BMIM-MS displays a peak around 625 cm^−1^ and a broader absorption peaked around 658 cm^−1^, which are likely due to the MS anion. On heating, at 260 K, both lines narrow, and the higher frequency absorption drastically shifts to lower frequencies (around 645 cm^−1^). On further heating, around 345 K there is a general broadening of the lines, the one at higher frequency shifts to about 655 cm^−1^, and the spectrum resembles the one measured at the lowest temperature. These features could be the signature of the so called cold-crystallization at 260 K—namely, a transition to a solid state, followed, upon further heating, by a transition to a liquid state at 340 K [46]. Indeed, this hypothesis is further supported by the spectra measured when cooling down to 160 K (reported in Supplementary Materials), which do not display any solidification, rather suggesting the occurrence of a glass transition. Equivalent evolution has also been reported by IR spectroscopy on other ionic liquids [1,2,47,48].

**Figure 6:**
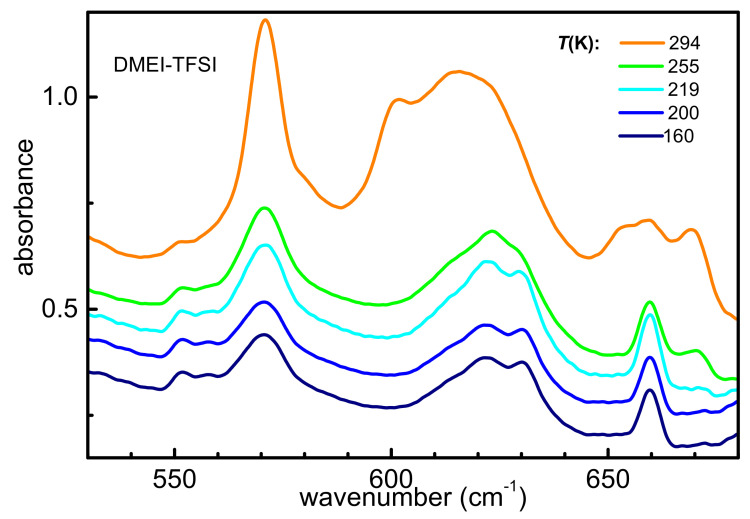
Absorbance of DMEI-TFSI measured on heating between 550 and 700 cm^−1^. The curves are vertically shifted for reasons of clarity.

Figure 6 reports the infrared absorption spectrum of DMEI-TFSI measured upon heating up to 320 K in the frequency range between 550 and 680 cm^−1^. As already reported by both computational and experimental research [2,41], this spectral range contains the markers of the two TFSI anion conformers: a narrow shoulder around 602 cm^−1^ and a broad band around 655 cm^−1^ are usually ascribed to the cis conformer, while a very intense peak around 618 cm^−1^ is attributed to the most energy-favored trans rotamer. As displayed in Figure 6, at the lowest temperature one can observe several absorptions: one at 570 cm^−1^, which is usually attributed to the superposition of absorptions due to both anion conformers [2,41], and one around 620 cm^−1^ with a high frequency shoulder and a narrow peak around 660 cm^−1^. On heating above 290 K, one observes a general narrowing of the lines and the appearance of a shoulder at 602 cm^−1^ and a broad band around 655 cm^−1^, which are likely due to the appearance of cis-TFSI. In this framework the lines at 620 and 660 cm^−1^, which are present in the whole temperature range, are respectively attributable to the trans conformer of the anion and to a cation vibration. These modifications are likely linked to the occurrence of melting around 290 K, with both conformers present in the liquid phase and only the most stable trans TFSI detectable in the solid phase. In turn, on cooling from room temperature, the infrared absorption spectrum of DMEI-TFSI (See Supplementary Materials) presents around 260 K a reversed transition, likely due to the occurrence of solidification.

#### 3.2.2. Intramolecular Interactions

**Figure 7:**
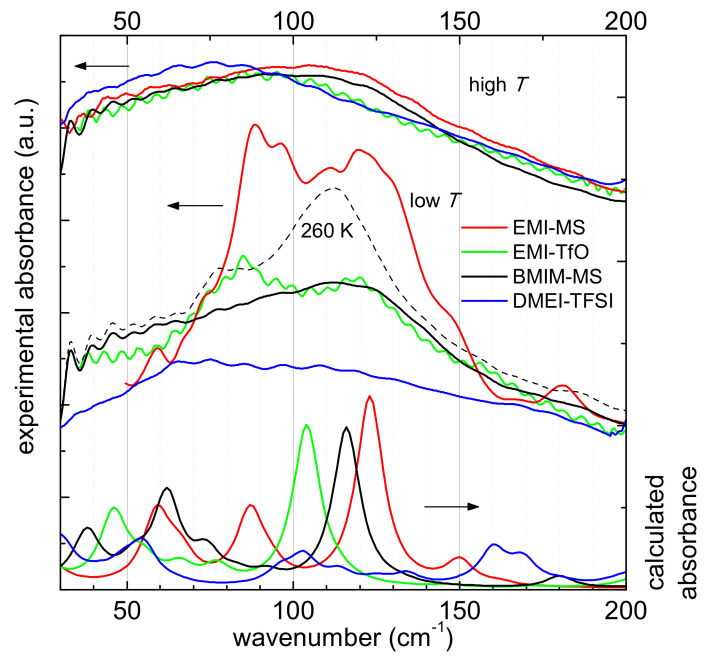
Far infrared experimental spectra of the samples DMEI-TFSI (blue lines), EMI-TfO (green lines), EMI-MS (red lines) and BMIM-MS (purple lines) measured at the highest temperature (top) and at 160 K (middle). For BMIM-MS the spectrum measured at 260 K is also shown (black dash line). The calculated spectra for all four ionic couples are presented in the bottom part of the figure.

The observations reported so far allow the assessment of the different phases experienced by the studied ILs in the measured temperature range; the following study of the temperature dependence of the far infrared spectrum of the samples provides information about the intramolecular interactions occurring in the different phases. Figure 7 reports the infrared absorption spectrum measured in the frequency range between 40 and 200 cm^−1^ for the four ILs at three different temperatures, which have been selected to take into account the different phases described above.

At the highest temperature, all samples are liquid, and their spectra are characterized by a broad feature peaked below 130 cm^−1^ (Figure 7). This low energy absorption appears at 70 cm^−1^ for DMEI-TFSI, around 90 cm^−1^ for EMI-TfO, and around 110 cm^−1^ for EMI-MS and BMIM-MS. It is likely due to the intermolecular interaction, mainly composed by dispersion forces [27,28,29,30], occurring within the ionic liquid and visible in this frequency range as a broad peak. It is worth noting that in the spectra measured at room temperature, the frequency of this feature seems to be affected by the anion present in each liquid, with the less interacting TfO inducing a peak at a lower frequency than the maximum observed in the ILs having the stronger interacting MS anion.

At the lowest temperature, the EMI-MS sample is solid (see above discussion), and its spectrum displays two narrower and well-developed absorptions at 90 and 120 cm^−1^ instead of the broad feature observable at room temperature around 110 cm^−1^. A similar behavior is also displayed by the sample EMI-TfO, which has the same cation of EMI-MS but a different anion (Figure 7) and whose spectrum at the lowest temperature, in the solid phase, is more structured than at room temperature; indeed, instead of the broad feature peaked at 110 cm^−1^, it is dominated by two narrower peaks, with maxima respectively at 85 and 120 cm^−1^.

These low frequency absorptions observed at low temperature for both EMI-based samples resemble the features reported in ammonium-based PILs having either MS or TfO as anion [8,9,11,21,24,25,26] and attributed to the dispersion forces (around 80 cm^−1^) and to the highly directional NH-anion interaction (around 50 and 110 cm^−1^) [9,21,22,24,25,26,49,50,51,52] typical of the hydrogen bond for this family of protic liquids. As a result, for the presently reported EMI-TfO and EMI-MS, we assign the feature at the highest frequency, i.e., around 120 cm^−1^, to the directional intermolecular interaction, similar to the one observed between the cationic NH group and the O atom of the anion. In the present case, it is likely due to the interaction between the SO_3_^−^ group of the anion and a H atom belonging to the cation, along the shortest O···H(-C) distance. Indeed, as reported in the computational results section, the shorter distances between H (cation) and O (anion) atoms are typical of moderate hydrogen bonding [42]. Moreover, the comparison with the calculated spectrum also suggests that the higher frequency peak could be due to the stretching of the C-H bond along the direction of the shortest O···H(-C) distance. The absorption curves generated from the computational results for these two samples show well-developed peaks between 100 and 125 cm^−1^ (see lower part of Figure 7): a visual inspection of the movements of the ions indicates that the peak around 125 cm^−1^ corresponds to the stretching of the C-H bond along the direction of the shortest O···H(-C) distance. For the sake of completeness, we also compared the absorbance spectra calculated by means of the ωB97X-D functional, dispersion forces, a polarizable medium, and a diffuse basis set (6–31++G**) (see Supplementary Materials Figures S3 and S4). All bands calculated by the latter method are displaced towards lower frequencies with respect to the experimental spectra and the absorbance spectra calculated at the ωB97X-/6-31G** level.

The sample BMIM-MS, as previously shown, in the considered temperature range displays three different phases:(i) it is likely to be in its glass state at the lowest temperature, (ii) to undergo a cold-crystallization around 260 K, (iii) followed upon further heating by a transition to a liquid state at 340 K. In Figure 7, in addition to the curve measured at the highest and lowest temperature, the spectrum of the sample in the solid state (at 260 K) is also reported. One can observe that the spectrum of the sample BMIM-MS measured at 160 K (Figure 7) does not display a remarkable difference compared with the highest temperature one, except for the appearance of a broad shoulder around 120 cm^−1^, superimposed on the broad absorption already detected at room temperature. This shoulder is more intense in the spectrum measured at 260 K and, similar to the case of EMI-containing samples, the comparison with the calculated spectrum (Figure 7) suggests that this higher frequency shoulder could be due to the stretching of the C-H bond along the direction of the shortest O···H(-C) distance.

In contrast to the previously reported ILs, for the DMEI-TFSI sample, the broad band centered around 70 cm^−1^ shows no temperature dependence (Figure 7) since the spectra measured at the highest temperature and at the lowest temperature, where it is solid, as well as at intermediate temperatures, are similar. Moreover, for this liquid, the calculated spectrum does not display the well-developed absorptions line in the frequency range between 90 and 150 cm^−1^, which are observable in the spectra of the other samples and are attributed to the stretching of the C-H bond along the direction of the lowest O···H(-C) distance.

In order to obtain information about the changes occurring in the molecular interactions and particularly in the hydrogen bonding involved in the different phases, a quantitative analysis of the temperature dependence of the FIR spectra was carried out, by means of a well-established method previously used to investigate [8,21] the competition between hydrogen bonding and the dispersion forces as a function of the temperature in protic ionic liquids. Using this model, one deconvolutes the observed absorbance into several contributions and, on the basis of the DFT calculated vibrations, one assigns these vibrational bands to the stretching (at higher frequency) and bending (at lower frequency) modes of the hydrogen bond superimposed on a vibrational band, with frequency ranging between these two, that describes the dispersion interactions between the anion and the cation. In particular, in agreement with the considered model used [8,21], we define:

(1) $$r=\frac{I_{Hbond}}{I_{disp.}}$$

where *I_disp_* indicates the integrated IR intensity of the band describing the configurations dominated by the dispersion interactions, and *I_Hbond_* indicates the integrated IR intensity of the band describing the configurations dominated by the hydrogen bonding [8,11,21] after subtracting a background. The ratio between the intensities of the vibrational bands corresponding to the two configurations provides the equilibrium constants, whose temperature variation can be described by the van’t Hoff relation:

(2) $$\ln\left(r\right)=-\frac{1}{T}\frac{\Delta H}{R}+\frac{\Delta S}{R}+c$$

The slope of the linear regression of ln(*r*) vs. 1/*T* provides the value of the difference in energy between the H-bonded and the dispersion-governed configurations [8,11]. This analysis was conducted in the temperature range where the samples were in their liquid phase, as the van’t Hoff equation is valid in this limit.

In particular, the absorbance spectrum measured on heating for the studied samples in the frequency range between 40 and 270 cm^−1^ was fitted by means of Lorentzian peaks. The calculated absorbance modes for EMI-TfO, EMI-MS and BMIM-MS are respectively centered at 118, 123, and 112 cm^−1^ for O···H stretching modes, while the bending modes appear at 77, 76, and 75 cm^−1^ (see Table 2). The bands due to dispersion-governed configurations are centered between 95 and 100 cm^−1^. For sample DMEI-TFSI, this analysis cannot be carried out since there is no clear evidence of the presence of hydrogen bonding either in the experimental spectra or in the calculated spectrum.

**Figure 8:**
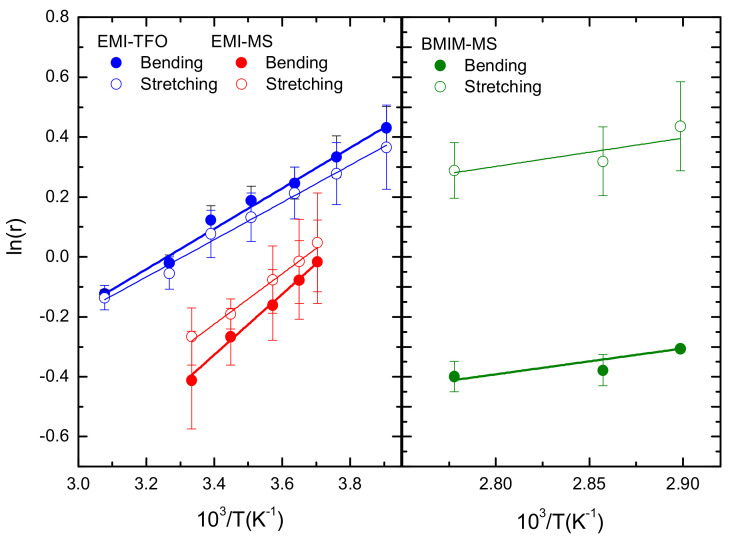
Logarithm of the ratio of intensities of hydrogen-bonding band to the dispersion-governed configurations band and best fit lines, as a function of temperature. EMI-TfO: blue circles;EMI-MS: red circles; BMIM-MS: green circles. Full symbols refer to ratio obtained considering the bending modes for the hydrogen bond-governed configurations, open symbols refer to ratio obtained considering the stretching modes.

The plots of ln(*r*) vs. 1/*T*, with $r=\frac{I_{Hbond}}{I_{disp.}}=\frac{I_{bending\left(stretching\right)}}{I_{disp.}}$, for the spectra measured in the liquid state of the three samples are reported in Figure 8.

The values of the average energy obtained by the best fit are reported in the Table 2. It is worth noting that, for all samples, the resulting van’t Hoff slope indicates that the H-bonded ion pairs are favored in energy over the dispersion-interaction-dominated pairs by about 5 kJ/mol for the TfO-based sample and ~8 kJ/mol for the sample with the MS anion.

## 4. Discussion

As discussed in the Introduction, the far infrared modes mostly probe the intermolecular interactions, and indeed the broad feature detected for all the measured liquids and peaked between 70 and 90 cm^−1^ is attributed to the dispersion forces. This feature presents a maximum at higher frequency for the more bonded couple, as demonstrated by the comparison of the two liquids sharing the same EMI cation. Indeed, this feature peaks at 90 cm^−1^ for the moderately bonded EMI-TfO and at 110 cm^−1^ for the more bonded EMI-MS couple.

At higher frequency—namely, around 120 cm^−1^—a narrower peak, which in EMI-MS, EMI-TfO, and BMIM-MS becomes well defined at the lowest temperatures, is attributed to the weak hydrogen bonding stretching along the shortest CH···O direction. This attribution is based on the comparison with the calculated spectra, which predicts a peak between 100 and 125 cm^−1^ (Figure 7), corresponding to the stretching of the C-H bond along the direction of the shortest O···H(-C) group. Indeed, for all samples, the lowest energy configurations obtained by DFT calculations show that the closest distance between one of the cation H atoms, linked to a C atom, and the closest anion oxygen atom is smaller than 2.48 Å, an interatomic distance typical of moderate to weak hydrogen bonding [42].

The results obtained for the lowest energy configurations provide a picture that does not rule out the occurrence of an anion–π interaction within these systems [53,54,55]. Indeed, this noncovalent interaction between an anion and the region above the plane of an electron poor aromatic ring has been reported to occur in imidazolium-based ILs [53,56]. In general, it is often observed in concert with H-bonding [53], which allows the coordination of the anion to the plane of the ring [54]. However, it must be pointed out that the absorption bands observed in the presently reported IR spectra around 120 cm^−1^ are typical of hydrogen bonding frequencies, as also confirmed by the DFT calculated modes.

Experimentally, one observes a broad absorption due to hydrogen bonding stretching and bending in the far infrared in the case of EMI-MS, EMI-TfO, and BMIM-MS, while there are no such bands in the DMEI-TFSI ionic liquid (Figure 7). Moreover, the absorption curves generated from the calculated vibrational frequencies and intensities show peaks between 100 and 125 cm^−1^, corresponding to the stretching of the hydrogen bond for the first three samples. On the contrary, DMEI-TFSI does not display a clear feature attributable to hydrogen bonding (see Figure 7). A detailed examination of the calculated values indicates that the hydrogen bonds of the four ionic couples present differences in the distances between the H and O atoms, as well as in the angle formed by the CHO atoms (see Table 1). Indeed, the DMEI-TFSI liquid presents the longest calculated distance between H and O; moreover, for EMI-MS, EMI-TfO, and BMIM-MS, the CHO angle ranges between 136 and 166°, so that the three atoms are relatively aligned, while for DMEI-TFSI, the CHO angle is 122°, implying that the O atom is misaligned with the CH direction.

The agreement between the measured and calculated spectra further confirms that the use of the ωB97X-D functional paired with the presence of a polar solvent and empirical dispersion forces is particularly suited to address a good description of the hydrogen-bonding bands in the far infrared range [8].

According to the criteria for Hbonds adapted from Steiner and Jeffrey [5,42,57], the liquids with MS or TfO as anion have the characteristic of a moderate interaction (H···O distance between 1.5 and 2.2 Å, bond angle larger than 130°), while DMEI-TFSI present values typical of a weak interaction (H···O distance > 2.2 Å and bond angle between 90 and 130°). This observation suggests that the occurrence of the stretching band of the hydrogen bonding in the FIR range requires at least the occurrence of a moderate interaction, i.e., a considerable alignment of the atoms forming the bond and a sufficiently short distance between them. In particular for the DMEI-TFSI, the presence of a weaker interaction is not detected by infrared measurements. Moreover, by means of a quantitative analysis of the temperature dependence of the infrared spectrum, we obtained an evaluation of the average energy difference between the dispersion-interaction-dominated and H-bonded configurations. These values are about 5 kJ/mol for the TfO-based sample and about 8 kJ/mol for the samples having a common MS anion (see Table 2). These results confirm that the hydrogen bond dominates at low temperatures, in agreement with previous analysis conducted on other ammonium-based protic ionic liquids [8,11,21], even though the values obtained for the energy difference are lower in the presently investigated samples. Indeed, the previously reported values for energy difference between the H-bonded and dispersion-governed configurations range up to 34 kJ/mol^−1^, as obtained by Fumino at al. [21] for trihexylammonium triflate (THA-TfO), confirming that for the presently studied IL the H bond is weaker than the one present in PILs, i.e., the +N-H···O3S^-^ hydrogen bond. It is worth noting that the MS-based sample (EMI-MS) displays a higher value for the difference in the average energy between the H-bonded and the dispersion-governed configurations compared with EMI-TfO, in agreement with the reinforcing of the hydrogen-bonding interaction by the substitution of the TfO anion with the stronger MS.

Moreover, the present measurements clearly show the domination of the H bond at lower temperatures, as the peak ascribed to the stretching mode of the C(H)-O bond became evident in the spectra measured at lower temperatures, and particularly when the samples are in their solid state. This is clearly visible for BMIM-MS (Figure 7), whose spectrum, measured on heating, at 260 K, i.e., entering the solid state, displays distinctly the narrow peak around 120 cm^−1^, which is otherwise less evident at lower temperature in the glassy phase.

It is worth noticing that the crystal structure of solid EMI-TfO has been reported [45] to be orthorhombic, with the ions packed in the crystal lattice via three weak interionic C–H···O hydrogen bonds, which connect the cations to the anions in the “ab”plane, forming a layered structure [45]. Moreover, good agreement can be noted between the shortest H···O distance calculated in the present work and that reported in Reference [45] (2.23 vs. 2.24 Å). The better CH···O alignment reported in Reference [45] in the crystalline phase (149°) with respect to that calculated by us in a simulated polarizable medium (≈137°) supports the fact that the hydrogen-bonding infrared absorption bands are much better identified at low temperatures in the solid phase than at high temperatures in the liquid state. Indeed, as discussed above, the alignment of the atoms is fundamental to detect the stretching modes of H bonds, and in the ordered solid phase, this is better achieved than in the more disordered liquid or glass phase. It must be noted, however, that hydrogen bonding is present at all temperatures, and indeed, the analysis to calculate the energy difference is performed in the liquid state.

The measurement of the FIR spectra as a function of the temperature in a wide temperature range below room temperature allows the clear detection of the possible occurrence of even moderate hydrogen bonding, overcoming possible uncertainty deriving from the mere analysis of room temperature spectra, where a broad band is usually observed due to the superposition of dispersion forces and possible H bonding. In particular, for imidazolium-based ILs, it was suggested that this band is practically unchanged even if the acidic C(2)-H is suppressed [27,28]. The present measurements show that below room temperature the FIR spectra present clear modifications, i.e., show the presence of the H-bonding stretching band when a strong to moderate interaction of this kind is present. In this case, the use of a model that takes into consideration contribution both from the dispersion forces and from the hydrogen bonding, instead of a simple superposition of the bending and stretching hydrogen bonding [29], allows the detection of changes with temperature and the calculation of the average energy difference between the dispersion-interaction-dominated and H-bonded configurations.

## 5. Conclusions

In this work, we present a combined theoretical and experimental approach to predict and detect the occurrence of less intense hydrogen bonding in aprotic ionic liquids. The DFT calculations were performed using the ωB97X-D functional and considering both the empirical dispersion corrections and the presence of a polar solvent. The lowest energy configurations of the ion pairs were identified, and their absorption curves were generated starting from the calculated vibrational frequencies and intensities. The considered ionic liquids were all imidazolium-based with anions of different bonding ability. For all the liquids, the lowest energy configuration of the ionic couple present H atoms of the cation, directly bonded to C atoms that are close to O atoms of the anions; however, the hydrogen-bonding stretching band is present only in the calculated IR spectrum of ILs with strongly attracting anions, such as MS or TfO. The calculations agree with the measured infrared spectra and their temperature dependence. In particular, the hydrogen-bonding stretching band is clearly detected in those liquids where it was computationally predicted, and the analysis of the spectra allows the detection of changes with temperature and the calculation of the average energy difference between the dispersion-interaction-dominated and H-bonded configurations, both playing central roles in the IL unique properties.

## Figures and Tables

**Table 1:** Distance between the closest H atom of the cation and O atom of the anion and CHO angle in various ion pair calculated by DFT by the ωB97X-D functional and considering the presence of a polar solvent.

Ion Pair	(H-O) (Å)	Angle(C-H-O) (°)
EMI-MS	2.08	152.2
BMIM-MS	2.10	166.1
EMI-TfO	2.23	136.6
DMEI-TFSI	2.48	122.0

**Table 2:** Calculated ΔH obtained from the linear fit regression of ln(*r*) vs. 1/T.

Sample	ΔH _bending_ (kJ/mol^−1^)	ΔH _stretching_ (kJ/mol^−1^)
EMI-TfO	5.6 ± 0.2	5.2 ± 0.3
EMI-MS	8.4 ± 0.4	7.0 ± 0.4
BMIM-MS	7.2 ± 2.3	7.9 ± 4.0

## Data Availability

The data presented in this study are available on request from the corresponding author.

## Supplementary Materials

The following are available online at https://www.mdpi.com/article/10.3390/ijms22116155/s1, Figure S1: Absorbance of BMIM-MS between 600 and 680 cm^−1^ measured upon cooling, Figure S2: Absorbance of DMEI-TFSI between 530 and 680 cm^−1^ measured upon cooling, Figure S3: Comparison of the experimental absorption spectrum of EMI-MS measured at 140 K with the absorbance spectracalculated by DFT at the ωB97X-D/6-31G** and ωB97X-D/6-31++G** levels, Figure S4: Comparison of the experimental absorption spectrum of EMI- TfO measured at 111 K with the absorbance spectracalculated by DFT at the ωB97X-D/6-31G** and ωB97X-D/6-31++G** levels, Figure S5: Comparison of the experimental absorption spectrum of BMIM-MS measured at 300 K with the absorbance spectrum calculated by DFT at the ωB97X-D/6-31G** level, Figure S6: Comparison of the experimental absorption spectrum of DMEI-TFSI measured at 300 K with the absorbance spectrum calculated by DFT at the ωB97X-D/6-31G** level, Table S1: Calculated infrared vibrational frequencies and intensities of the four ionic couples.
