# Supplementary material for: Evidence of the CH···O HydrogenBonding in Imidazolium-Based Ionic Liquids from Far-Infrared Spectroscopy Measurements and DFT Calculations
Source: Int J Mol Sci. 2021 Jun 7;22(11):6155. doi: 10.3390/ijms22116155 (PMC8201385; doi:10.3390/ijms22116155)
Supplement: Supplementary file 1 [file ijms-22-06155-s001.zip › ijms-1237284-supplementary.pdf]

International Journal of Molecular Sciences

MDPI

Evidence of the CH···O hydrogen bonding in imidazolium based ionic liquids from far-infrared spectroscopy measurements and DFT calculations

Oriele Palumbo [1], Adriano Cimini [1], Francesco Trequattrini [2, 1,*], Jean-Blaise Brubach [3], Pascale Roy [3] and Annalisa Paolone [1]

1 CNR-ISC, U.O.S. La Sapienza, Piazzale A. Moro 5, 00185 Roma, Italy ; oriele.palumbo@roma1.infn.it; annalisa.paolone@roma1.infn.it; cimini.1233261@studenti.uniroma1.it
2 Physics Department, Sapienza University of Rome, Piazzale A. Moro 5, 00185 Roma, Italy ;
3 Synchrotron SOLEIL, AILES Beamline, L'Orme des Merisiers Saint-Aubin, BP 48, 91192 Gif-sur-Yvette Cedex, France; pascale.roy@synchrotron-soleil.fr; jean-blaise.brubach@synchrotron-soleil.fr
* Correspondence: francesco.trequattrini@roma1.infn.it

Table S1. Infrared vibrational frequencies (ω in cm^{-1}) and intensities (I in km/mol) of the four ionic couples calculated at the ωB97X-D level of theory with the 6-31G** basis set and a polar medium.

EMI-MS		BMIM-MS		EMI-TfO		DMEI-TFSI	
⚜	I	⚜	I	⚜	I	⚜	I
11	17	9	12.67	21	7.45	26	5.35
26	3.06	37	8.9	27	0.39	30	4.72
58	11.93	51	3.47	45	12.3	37	0.44
65	4.49	61	15.03	54	3.63	41	1.22
86	12.18	73	4.96	65	2.92	48	3
92	2.07	82	1.36	76	2.95	54	6.48
122	32.25	91	1.62	103	27.46	64	0.41
149	4.14	103	0.04	113	0.83	95	2.33
161	0.52	115	27.12	145	0.34	102	4.87
226	0.53	131	0.4	171	0.25	113	2.21
236	2.2	179	1.99	207	3.61	124	1.1
240	0.01	209	1.12	214	5.63	133	1.92
304	1.32	247	0.07	236	1.2	150	0.18
332	1.59	248	1.79	240	1.81	159	6.04
334	2.07	295	0.74	313	2.85	168	4.11
407	1.23	316	3.53	320	4.06	179	0.57
431	0.9	333	1.79	345	0.65	197	0.64
513	41.91	334	2.35	348	1.76	208	5.6
514	41.22	393	1.73	400	0.89	212	3.52
543	129.96	441	4.92	442	0.93	245	0.18
618	1.6	498	5	505	42.12	248	0.47
645	11.6	513	39.61	507	42.06	276	2.64
677	49.19	515	42.67	572	14.77	283	15.03
725	6.83	546	122.2	574	12	297	5.95
770	51.29	629	2.77	617	18.52	301	8.17
778	117.9	642	24.91	623	306.09	325	1.89
815	8.2	679	21.44	643	28.05	330	1.5
891	0.04	721	0.62	670	31.15	334	1.79
933	76.44	774	40.36	725	6.46	346	0.34
977	6.63	778	106.43	760	45.34	349	0.84

998	0.84	789	25.64	776	11.04	399	15.43
999	1.45	831	9.91	818	7.65	418	2.24
1050	248.46	869	36.6	866	67.42	423	18.28
1056	25.18	904	4.06	886	0.37	497	197.57
1067	2.53	946	9.54	978	5.94	499	1.65
1122	14.46	975	6.97	1035	288.9	525	23.87
1127	11.32	995	0.93	1056	3.09	548	6.73
1135	9.77	999	1.12	1068	3.88	560	7.26
1152	1.91	1001	1.01	1121	12.27	568	67.73
1167	16.73	1051	185.3	1126	10.06	575	99.06
1200	249.66	1053	88.09	1136	5.33	587	251.91
1219	360.88	1064	1.59	1153	2.66	600	0.06
1234	530.87	1091	2.45	1169	14.34	649	184.73
1288	4.55	1124	6.44	1209	115.84	652	6.81
1330	0.9	1136	4.84	1226	248.47	694	26.34
1382	25.17	1150	4.19	1228	215.27	736	6.07
1399	19.38	1156	14.48	1258	574.6	741	91.69
1404	8.56	1171	0.46	1265	123.2	754	13.56
1433	10.91	1212	474.01	1274	528.54	775	63.52
1451	3.2	1218	223.18	1287	11.98	782	3.84
1463	6.73	1232	451.33	1329	1.21	799	60.7
1468	7.08	1237	23.61	1398	23.15	830	5
1469	5.25	1282	3.67	1405	6.05	890	0.25
1490	8.63	1325	5.91	1433	11.22	985	7.69
1490	6.91	1342	0.43	1452	4.72	1009	1.06
1498	15.71	1381	7.61	1470	7.36	1053	600.1
1499	13.5	1382	25.06	1490	4.7	1061	22.79
1513	12.12	1394	7.91	1495	6.56	1093	2.69
1520	13.06	1412	1.73	1498	16.71	1107	15.96
1640	63.19	1416	14.03	1500	15.5	1127	2.08
1650	71.26	1436	5.43	1520	12.17	1134	14.14
3074	17.04	1454	13.15	1520	16.75	1142	596.99
3100	3.1	1467	5.05	1644	40.48	1148	706.98
3100	29.39	1468	5.16	1651	95.57	1161	7.04
3129	16.14	1474	7.64	3077	17.14	1175	42.68
3162	5.42	1484	17.43	3101	23.02	1246	590.61
3175	23.73	1485	11.14	3131	15.29	1246	12.29
3194	13.72	1491	9.82	3164	9.24	1250	131.27
3204	3.56	1497	3.08	3177	19.02	1255	446.23
3207	4.44	1507	5.94	3197	9.41	1259	77.25
3213	2.87	1513	17.37	3203	1.89	1262	296.01
3215	4.37	1518	28.84	3216	2.01	1267	42.58
3234	340.38	1522	8.01	3311	145.83	1279	31.88
3325	18.12	1649	32.98	3335	19.38	1333	181.55
3344	10.73	1654	102.26	3352	9.54	1335	28.23
		3056	49.28			1345	629.24
		3061	32.81			1402	28.64
		3077	21.82			1413	16.65
		3098	3.22			1428	9.66
		3105	32.98			1440	14.31
		3109	19.2			1443	6.14

	3121	9.11	1463	51.77
	3134	37.16	1475	8.2
	3137	88.55	1485	5.24
	3150	52.2	1499	18.86
	3188	3.98	1500	2.18
	3206	3.26	1505	21.93
	3206	5.44	1512	7.74
	3211	5.26	1530	29.3
	3225	5.24	1589	17.66
	3227	300.16	1610	132.58
	3337	47.46	1663	28.38
	3343	4.69	3082	11.24
			3097	2.7
			3111	19.21
			3130	16.85
			3170	6.56
			3174	19.29
			3182	0.71
			3192	16.43
			3200	1.21
			3209	2.34
			3222	1.52
			3339	18.07
			3360	12.42

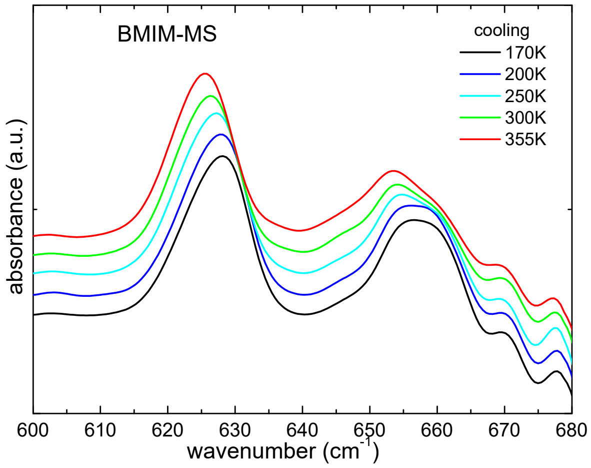

Figure S1. Absorbance of BMIM-MS between 600 and 680 cm^{-1} measured upon cooling.

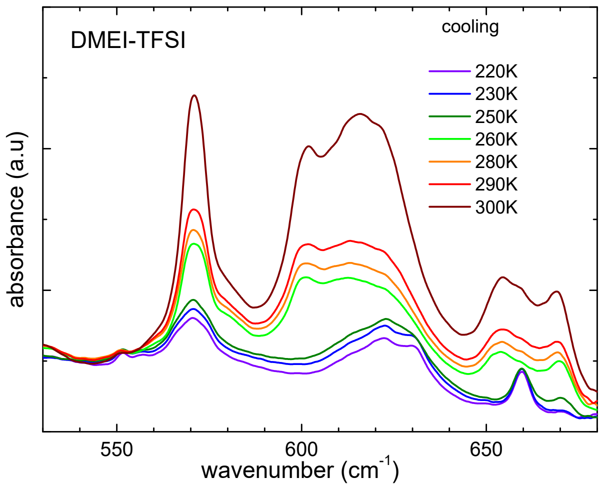

Figure S2. Absorbance of DMEI-TFSI between 530 and 680 cm^{-1}measured upon cooling.

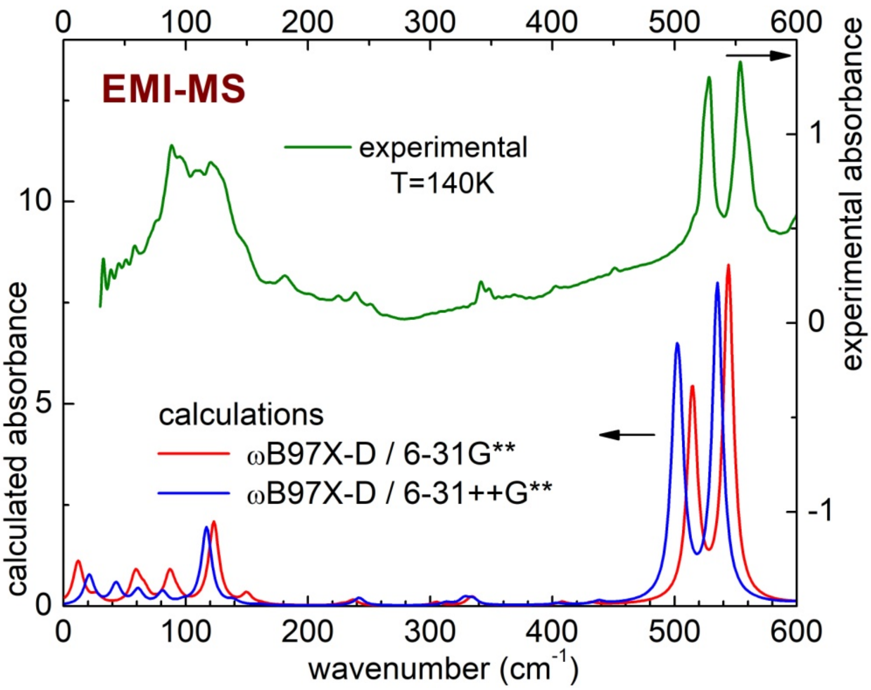

Figure S3. Comparison of the experimental absorption spectrum of EMI-MS measured at 140 K with the absorbance spectra calculated by DFT at the ωB97X-D/6-31G** and ωB97X-D/6-31++G** levels.

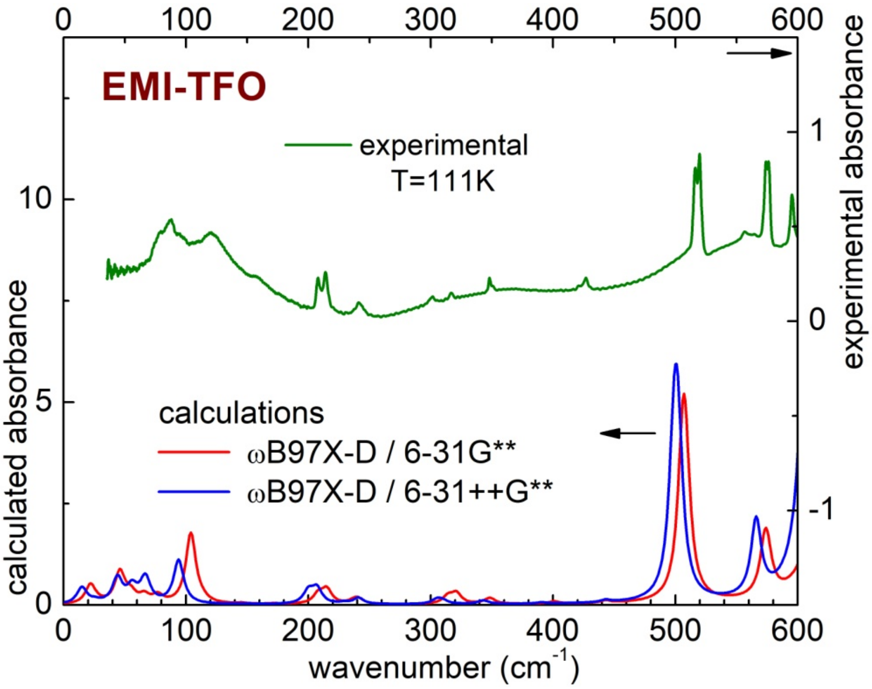

Figure S4. Comparison of the experimental absorption spectrum of EMI-TFO measured at 111 K with the absorbance spectracalculated by DFT at the ωB97X-D/6-31G** and ωB97X-D/6-31++G** levels.

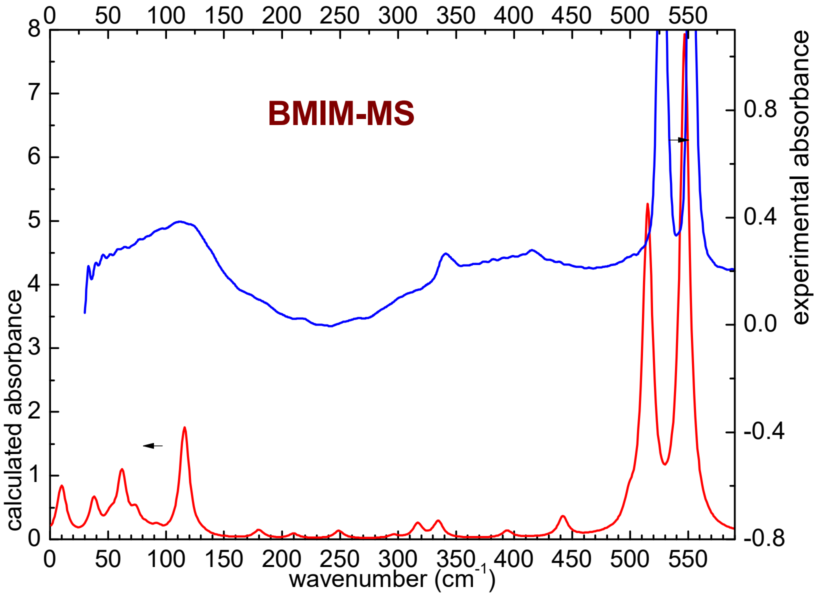

Figure S5. Comparison of the experimental absorption spectrum of BMIM-MS measured at 300 K with the absorbance spectrum calculated by DFT at the ωB97X-D/6-31G** level.

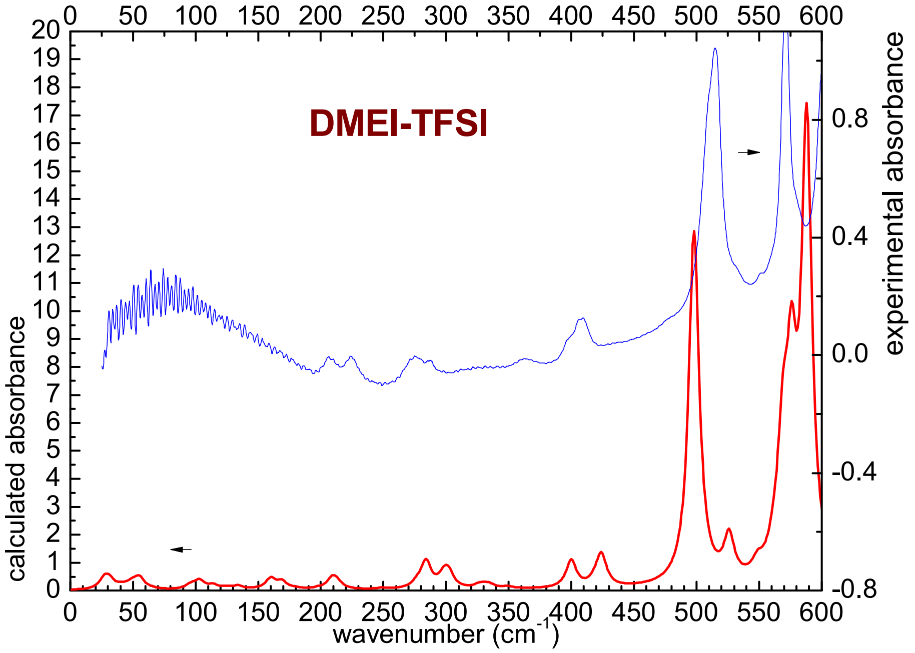

Figure S6. Comparison of the experimental absorption spectrum of DMEI-TFSI measured at 300 K with the absorbance spectrum calculated by DFT at the ωB97X-D/6-31G** level.
